# The barriers to access healthcare services by people with disabilities: Policymakers’ perspectives

**DOI:** 10.4102/ajod.v15i0.1790

**Published:** 2026-06-25

**Authors:** Hileni N. Niikondo, Sipho W. Mkhize

**Affiliations:** 1 School of Nursing and Public Health, Faculty of Health Sciences and Veterinary Medicine, University of Namibia, Windhoek, Namibia; 2 School of Nursing and Public Health, College of Health Sciences, University of KwaZulu-Natal, Durban, South Africa

**Keywords:** access, barriers, disabilities, healthcare service, perspectives, policymakers

## Abstract

**Background:**

Accessibility to healthcare is a fundamental human right aimed at facilitating better health outcomes for people with disabilities. However, the persistent gap between policy intents and actual implementation results in poorer health outcomes for people with disabilities, which is against universal health coverage.

**Objectives:**

This study explored policymakers’ perspectives on barriers to accessibility to healthcare services for people with disabilities.

**Method:**

Through purposive sampling, qualitative, descriptive, exploratory research was conducted among five policymakers in a rural constituency. Each participant signed an informed consent form. The face-to-face interview lasted 50–60 min and used validated semi-structured questions, audio recordings, and memos to capture data. Manual coding, employing an inductive approach and categorisation, was conducted, resulting in the formulation of themes.

**Results:**

Two main themes emerged: Systemic and structural challenges to inclusive healthcare, and training and attitudinal barriers to inclusive healthcare, highlighting transport and infrastructure barriers, as well as insufficient budget because of ineffective health policy implementation. A lack of training and discrimination were key issues reported.

**Conclusion:**

This study highlighted key obstacles related to weak policy enforcement, inadequate infrastructure, stigma, and funding issues, which impede accessibility to health services for people with disabilities in Namibia.

**Contribution:**

Understanding how policies are translated into practice is crucial for closing implementation gaps and enhancing healthcare access for people with disabilities.

## Introduction

Access to healthcare is a fundamental human right aiming to facilitate better health outcomes; however, people with disabilities’ access to healthcare services is jeopardised by various limitations, including physical, systemic, attitudinal, and policy-related barriers. According to the World Health Organization (WHO), a population of 1.3 billion people, which is about 16% of the global population, live with some form of disability and experience exclusion from essential healthcare services because of discriminatory practices, inaccessible settings, and inappropriate policies (McClintock et al. 2018; WHO 2023, 2025).

Globally, people with disabilities face considerable challenges in accessing healthcare services. They often experience higher rates of unmet health needs than the general population because of physical, financial, and systemic barriers (WHO 2022). In many low- and middle-income countries, the health needs of persons with disabilities and their families are not sufficiently covered because of limited resources and a lack of disability-inclusive policies (WHO 2022). Several studies highlight persistent barriers, including inaccessible health infrastructure, untrained staff, discriminatory attitudes, and a lack of assistive communication methods for individuals with visual or hearing impairments (Hashemi, Kuper & Wickenden 2020). Mental health stigma further limits access for individuals with psychosocial disabilities. The lack of coordination between disability policies and healthcare delivery contributes to fragmented care (Amadhila et al. 2024; Smythe et al. 2022). In Namibia, people with disabilities are deprived of access to information, communication, adequate infrastructure, and assistive technologies. Most of these obstacles are linked to weak policy enforcement, inadequate infrastructures, stigma, and funding issues, which impede accessibility to health services for people with disabilities in Namibia. Often, limited disability awareness among health professionals contributes to weak policy enforcement and implementation (Amadhila et al. 2024).

Although the Universal Health Coverage emphasised health for all, some barriers compromise the health outcomes of people with disabilities people with disabilities, which violates their rights (Cu et al. 2021; WHO 2023). Despite the existence of policies that advocate for preferential treatment and inclusivity, the practical implementation of these policies varies across settings and among stakeholders of people with disabilities. In Africa, people with disabilities are challenged by poverty, long distances, stigma, and transportation barriers, which impact the delivery of healthcare (Lagu et al. 2022). Often, people with disabilities are exposed to inappropriate healthcare, poor housing, a lack of access to safe water, and sanitation barriers (WHO 2025). In some African countries, despite the constitutional obligation for disability inclusivity, persons with disabilities do not have access to quality health services, leaving significant gaps in compliance with the Convention on the Rights of Persons with Disabilities (CRPD) (African Disability Forum 2020). Low- and middle-income countries differ in the way they formulate and implement policies, including disability laws. Various stakeholders interpret and implement disability-related laws differently, which creates discrepancies among settings and leads to barriers to healthcare access and societal exclusion for people with disabilities. People with disabilities should have access to healthcare services that are not only physically accessible but also culturally sensitive, adequately resourced, and responsive to their unique needs (Amado et al. 2015).

Nevertheless, inclusive policy frameworks and disability laws are documented as part of state efforts to improve access to healthcare for people with disabilities (African Disability Forum 2020). However, a persistent gap between policy intent and actual implementation results in people with disabilities being exposed to poorer policy implementations, which leads to unmet health needs, delayed treatment, and poorer health outcomes (Shumba & Moodley 2018).

Most countries have adopted disability-inclusive policies in line with the CRPD. However, a gap exists between policy availability and implementation because of challenges such as inadequate funding, insufficient monitoring, limited disability-specific data, and limited intersectoral collaboration (Muhammad 2024; Zhao & Wang 2021). Training healthcare workers in disability awareness and communication, community engagement, and integrating disability issues into mainstream health planning is vital to improving accessibility. The availability of inclusive infrastructure, reliable assistive technologies, and empowered caregivers also enhances access (Lagu et al. 2022; Zhao & Wang 2021). While most research focuses on people with disabilities and healthcare providers, studies exploring policymakers’ views are limited. Therefore, it is essential to understand policymakers’ perspectives on translating these policies into practice, which is crucial for closing these implementation gaps.

## Research methods and design

Research methods are forms of data collection, analysis, and interpretation that researchers use when proposing studies (Creswell 2014). In this study, the researchers employed a qualitative method because it is well-suited to capturing complex experiences, perceptions, and emotions, thereby gaining a deeper understanding of individuals’ experiences that cannot be measured or described numerically.

### Study design and setting

The research design is the plan for conducting the study, specifying the type of data to be collected and the methods used (Creswell 2014; Nieswiadomy & Bailey 2018).

A qualitative, descriptive, exploratory, and contextual study design was used to investigate the experiences of policymakers to investigate how policies address access to healthcare for people with disabilities in Omulonga constituency. Namibia is divided into 14 regions, which are further subdivided into 121 constituencies. The Ohangwena region consists of 12 constituencies, including Omulonga, where this study was conducted.

### Study population and sampling

A population refers to an entire group that meets the inclusion criteria and is suitable for the study (Nieswiadomy & Bailey 2018). Sampling is the process of selecting participants to represent the study population (Gray & Grove 2021).

The population was five government employees and one traditional authority disability coordinator. They represent a particular unit or programme that implements government policies at the constituency level, and their participation provides input to the law-making process through consultation, ensuring that the interests of the constituency are reflected in national legislation through regional representation. Policymakers should have at least 2 years of experience and have worked with laws and regulations related to disability affairs.

Participants were identified through purposive sampling. Purposive sampling is a non-probability technique in which participants are intentionally selected based on their ability to provide relevant and rich information for the study (Gray & Grove 2021). In this study, the selection focused on those who had experience of worked with disability issues in the constituency. Data saturation occurs when collecting fresh data no longer yields new insights or reveals new properties (Creswell 2014). Data saturation occurred at participant number four; however, to ensure representation of diverse perspectives, the researcher also interviewed a disability programme manager who serves at the regional level and occasionally conducts constituency visits.

### Data collection

Data collection is the process of gathering data through various techniques and contexts to address the study’s research questions (Nieswiadomy & Bailey 2018; Polit & Beck 2021).

A semi-structured interview guide, validated with assistance from a peer researcher and a supervisor, was used to explore policymakers’ perspectives on disability-related matters. Interviews were conducted in September 2023 for a week after the primary researcher received ethical approval and the necessary permissions to conduct the study. The primary researcher consults with the coordinator of disability affairs and the constituency counsellor for gatekeeper permission to conduct the interviews. The researcher introduces the research team and explains the study’s purpose to each participant before the interview. Each face-to-face interview was conducted at the participant’s workplace, in a quiet area, after the participant signed the consent form. Permission to use an audio recorder was obtained from each participant at the start of the interview, which lasted 50–60 min. Data collection tools, such as an audio recorder and field notes, were used to capture information and observations, respectively. Interviews were conducted in English, the country’s official language, and all participants communicated smoothly. Data saturation was reached when no new information emerged.

### Data analysis

Data were analysed using thematic analysis, which involved transcription, coding, categorisation, and theme development to identify patterns in participants’ experiences (Brink, Walt & Rensburg 2018). Audio recordings were transcribed verbatim to prepare for thematic data analysis, following an inductive approach (Braun & Clarke 2019; Creswell & Poth 2018). The researcher read through each transcript multiple times to gain a deeper understanding of potential ideas. The researcher conducted manual coding by identifying and highlighting key issues in each transcript related to the study objectives, grouping similar patterns in columns, and then categorising these patterns. Comparable categories were then used to develop themes, each with its own subthemes.

The researcher revisits all data for each theme to ensure that the themes accurately reflect the participants’ perspectives. Each final theme was developed through clear definitions and supported by illustrative quotes from participants. The report was written based on existing literature and participants’ accounts, and Grammarly was used to edit the text.

### Trustworthiness

Dependable and credible techniques were used, as emphasised by methodological authors (Creswell & Poth 2018; Korstjens & Moser 2018). Credibility was ensured through prolonged engagement, as the interviews lasted approximately 50–60 min. Triangulation was achieved by conducting interviews at multiple sites, and various tools, including voice recordings and field notes, were used to document the actions. Also, member checking was ensured through the validation of responses by participants. The transcripts were sent to participants to validate the accuracy of their views through feedback. Transferability was supported with a rich context, which explains the research process. To ensure dependability, an audit trail was maintained through the research transcripts and coding, which were reviewed by both the supervisor and the peer researcher. Conformability was achieved by grounding findings in data and clearly explaining the coding process. Authenticity was reflected in the fact that all participants’ views were fairly represented.

### Reflexivity

The primary researcher kept reflexive notes and discussed them with a peer researcher to ensure that assumptions did not influence the findings and that the analysis reflected participants’ responses (Korstjens & Moser 2018).

### Ethical considerations

Ethical clearance to conduct this study was first obtained by the corresponding author from the Biomedical Research Ethics Committee (Protocol reference number: BREC/00005244/2023) at the University of KwaZulu-Natal, and permission to conduct the study from the Namibian Ministry of Health and Social Services (Reference: 22/4/2/3). The researcher also obtained gatekeeper permission from the counsellor of Omulonga constituency before embarking on data collection. Each participant signed an informed consent before the interview. Participation was voluntary, with an option to withdraw without penalty if one felt uncomfortable during the process. All data were treated with strict confidentiality throughout the study. Participants’ identities were protected by using pseudonyms instead of their real names. Any identifying information was removed from transcripts and research records to ensure anonymity. Electronic data were password-protected, and hard copies were securely stored in a locked cabinet accessible only to the researcher.

## Results

### Socio-demographic characteristics

Most participants were male and older, and had sufficient exposure to policy-related matters affecting people with disabilities (see Table 1).

**TABLE 1:** Socio-demographic characteristics of participants.

Participant	Age group(years)	Gender	Years of experience	Government employee
PM_1	58–67	Male	25	No
PM_2	48–57	Male	12	Yes
PM_3	38–47	Female	4	Yes
PM_4	28–37	Female	3	Yes
PM_5	28–37	Male	5	Yes

PM, policymaker.

Government employees and traditional leaders characterise the participants’ socio-demographic characteristics. The participants’ age distribution reflects their actual ages at the time of data collection; however, the age groups (years) were used to ensure anonymity.

### Themes

Analysis of interview transcripts from policymakers revealed several interrelated barriers that hinder access to healthcare services for people with disabilities. Two key themes emerged, each supported by direct expressions from participants. Theme 1 focuses on systemic and structural challenges to inclusive healthcare, including transportation and infrastructure for healthcare access and inadequate policy implementation, as subthemes.

Theme 2 focuses on training and attitudinal barriers to inclusive healthcare, encompassing issues such as the impact of a lack of training on disability matters and the impact of stigma and discrimination on people with disabilities.

#### Theme 1: Systemic and structural challenges to inclusive healthcare

The ability to provide effective, inclusive healthcare services is hindered by several systemic and structural challenges that are beyond the control of healthcare providers.

**Transport and infrastructure for healthcare access:** To enable persons with disabilities to live independently, access to transportation for healthcare is key. However, people with disabilities face difficulties in physically reaching healthcare facilities because of a lack of transport services. Policymakers found that many people with physical or visual impairments in rural areas were unable to access transport that accommodates their needs. Public transportation is often unavailable, and the cost of private transportation is too high for many. This lack of accessible and affordable transportation hinders healthcare-seeking behaviour, negatively impacting health outcomes. These were narrated by participants as follows:

‘The lack of transport makes it difficult for them [*people with disabilities*] to reach their destination. Sometimes, they do not even have money to pay for that car.’ (PM_2)

‘Most of the time, these people want to travel, they must book a car, and they are going to pay as far as N$200 for taking them from the village to the hospital. So, it is costly, and it is difficult for them, especially those who are living in remote areas.’ (PM_5)

Consequently, a physical barrier is described by participants in terms of inaccessible infrastructure, which limits the ability of people with disabilities to navigate and use the healthcare facilities. Participants narrated that mobility within these facilities becomes a struggle because of a lack of user-friendly infrastructure. Participants raised concerns about the poor state of healthcare infrastructure, particularly regarding accessibility for people with disabilities. Healthcare facilities lack ramps and wide entrances, making it difficult for wheelchair users and people with mobility impairments to access services independently without their family members. The lack of inclusive design led to dependence and discouraged frequent visits to health services:

‘Sometimes even the narrowness is just not friendly, so that the wheelchair can fit. We always recommend people to build infrastructures that are environmentally friendly.’ (PM_5)

‘One thing that I have noticed about our health centres is that they are not built in a way that is sometimes accessible to persons using wheelchairs.’ (PM_3)

Equally, policymakers mentioned the need to improve the structure of sanitation facilities, noting that most health facilities do not accommodate people with disabilities. Toilets are often too small for wheelchairs, requiring the architects to integrate provisions for people with disabilities in their designs. As a result of the sanitation challenges, people with disabilities frequently stay away from the healthcare services to avoid discomfort:

‘Yes, specialised toilets are crucial, to be designed to meet the unique needs of children with disabilities for hygiene and ease of use.’ (PM_4)

‘Our focus should be on making all constructions inclusive, which means creating entrances and exits that cater to wheelchair users and those relying on crutches.’ (PM_2)

Inadequate policy implementation: Another challenge reported by participants is a systemic issue related to inadequate policy implementation, reflected in insufficient funding and non-compliance with policy frameworks. Policymakers in disability affairs reported that funding for disability-specific services and infrastructure remains very limited. They have expressed that poor budgetary planning is affecting service delivery, as there is often non-compliance with fiscal policies. The funding gap implies that disability issues are not considered necessary, resulting in a significant lack of provision of assistive devices:

‘We did not provide wheelchairs because the budget was not enough, and sometimes, we failed to identify those in need of wheelchairs.’ (PM_2)

‘It is a budget issue; they tell you they do not have money for that. I think the government needs to increase the budget allocated for persons with disabilities. Disability is mentioned in planning, but when the money comes, it’s not prioritised.’ (PM_5)

Although policymakers acknowledged the presence and use of supportive laws and policies to guide outreach programme planning and resource allocation, they also found barriers to implementing these policies because of inconsistent enforcement across service providers. They indicated a lack of awareness, weak enforcement mechanisms, and the absence of monitoring systems. This separation between policies and practices results in inconsistent and inadequate health service delivery to people with disabilities:

‘We have laws and policies, but they are collecting dust on our tables. We do not read the stipulations stated in policies to identify the remedial actions. And we fail to monitor the implementation, and secondly, we fail to evaluate whether that policy makes an impact [*blaming his office*].’ (PM_2)

‘I might not be familiar with the policy and what it says, but all I know is that everybody has the right to healthcare facilities. For example, when they go to the hospital, they do not need to pay. They have access to clinics. Accessibility is there.’ (PM_3)

A lack of policy implementation, as narrated by participants, results in unprioritised health services that create barriers hindering efficient service delivery to people with disabilities. Participants narrated a concern relating to the lack of prioritising persons with severe disabilities for treatment at healthcare facilities:

‘There is no specialised treatment for different disability categories, leading to challenges for those with visual impairments and those seeking general services. The queue is the same.’ (PM_2)

‘I think people with disabilities are struggling everywhere they are. Everything is difficult for them to get. So why don’t we just give them priority when it comes to health? Just imagine somebody who is already struggling to walk or who is sitting in a wheelchair, and so on. So, I think it is high time we prioritise them, whereby they come and queue to be attended to first to proceed.’ (PM_5)

Subsequently, policymakers expressed the need for collaborative arrangements to enhance service efficiency, reduce duplication of effort, and improve overall health service delivery for people with disabilities, especially those with multiple or severe impairments. They suggested a referral system that includes departments, non-governmental organisations (NGOs), and social service providers to improve access to healthcare for people with disabilities:

‘People should just work together and put more effort into helping people with disabilities, and things should not be done in isolation; they need to collaborate.’ (PM_1)

‘Major services, especially for visual impairments, require referral to district hospitals. Traveling to these locations requires energy and transportation. Equally, the involvement of stakeholders in the drafting and implementation of policies helps bridge the gap between legislation and practical application.’ (PM_2)

#### Theme 2: Training and attitudinal barriers to inclusive healthcare

Training and attitudinal barriers to inclusive healthcare services had an impact on people with disabilities’ access to health services in their context. This was expressed as follows:

The impact of a lack of training on disability matters: Participants highlighted that the lack of training on disability matters influences inclusive service delivery to people with disabilities. They emphasised the need to train healthcare providers to improve accessibility, noting that most have not received adequate training and therefore lack competence in treating and interacting with people with disabilities. As a result, people with disabilities are often misdiagnosed and discriminated against when seeking healthcare. Healthcare providers usually lack the knowledge and skills to communicate effectively in sign language and interpret treatment procedures for individuals with sensory disabilities. Healthcare providers also lack the skills to handle mental health patients:

‘I am just expressing the importance of giving information on disability, until such a time that everybody is educated on disability, very few people will understand the needs of people with disabilities. Also, the community at the villages, but even healthcare providers, general policymakers, parliamentarians, the councils, and the police drafters, need to be sensitised when it comes to disabilities.’ (PM_5)

‘The communication barrier is substantial. Even healthcare providers struggle to understand sign language, making it challenging for people who are hard of hearing to convey their health concerns. Visual impairments also pose challenges, particularly when attempting to comprehend written information. We need solutions that address these issues, perhaps even trained personnel to assist.’ (PM_2)

The lack of training in disability matters translates into another challenge, which impacts healthcare access to people with disabilities:

The impact of stigma and discrimination on people with disabilities: Negative attitudes and discriminatory behaviours among the healthcare providers were noted by participants, who narrated that some healthcare providers display negative attitudes and discriminatory behaviours when treating people with disabilities. Such attitudes contribute to feelings of exclusion, disempowerment, and mistrust in the health system. Likewise, family members do not disclose the presence of people with disabilities because of the fear of discrimination. This behaviour affects the ability to seek healthcare, resulting in delayed treatment:

‘This stereotype is among the society itself and is not exclusive to the healthcare providers; they might do this equally.’ (PM_5)

‘There is a need to have a specific programme focusing on the caregivers of people with disabilities. Why am I saying that? There are cases where a family has a person with disabilities, but they are not open. There are those cases of persons with disabilities who stay in the houses; they do not even go out.’ (PM_3)

## Discussion

This study aimed to investigate policymakers’ perspectives on access to healthcare services for people with disabilities. The study revealed several interrelated barriers that hinder access to healthcare services for people with disabilities, who are affected mainly by inequality because of social limitations. The crucial challenges mentioned are the unavailability of transport to support people with disabilities in accessing health services, infrastructures that are not in favour of people with disabilities, and a limited budget that hampers the policy’s implementation. As a result, efficient healthcare provision is hindered, limiting full access to healthcare. Based on these findings, the discussion will focus on the key themes and will be supported with literature.

Policymakers in this study mentioned systemic and structural barriers affecting people with disabilities in healthcare access. Participants found that the transport system remains a key obstacle, especially in rural areas, where disability-friendly public transport is hindered by poor road infrastructure, which restricts access to health services. The outcome aligns with findings from earlier studies, which underscore the importance of transport as a significant determinant of healthcare access for people with disabilities, leading to financial strain (Mitra et al. 2017; Soltani et al. 2019). Participants also mentioned the high cost of private transport, which worsens this problem as people with disabilities pay an exacerbated cost, which creates a financial barrier and prevents people with disabilities from seeking regular health services. Inclusive policy intervention is a facilitative approach that removes transport barriers and ensures access to healthcare. This implies that inclusivity, as a commitment to Universal Health Coverage, should focus on the ability of people with disabilities to access healthcare (African Disability Forum 2020).

Another critical barrier identified was the inappropriate design of healthcare facility buildings. Institutional documents highlight promising expectations for policy plans to improve and expand infrastructure for people with disabilities; however, these were hindered by limited policy implementation and budget constraints (Republic of Namibia 2016; WHO 2018). This study highlighted inaccessible infrastructure, including narrow entrances, a lack of ramps, and inaccessible restrooms, which made it physically difficult for wheelchair users to access services. Earlier studies also indicated non-compliance with policy promises to improve infrastructure, potentially to increase access to health services (Kuper & Hanass-Hancock 2020). Inaccessible sanitation structures made it difficult for people with disabilities to use the healthcare services in a dignified manner. These imply that collaborative planning for healthcare infrastructure is required to address these shortcomings.

This study found that there is often insufficient budget allocation for disability affairs. Participants reported a lack of funding for assistive devices, infrastructure, and sanitation suitable for people with disabilities. This finding is consistent with the conclusions of several global reports, which emphasise that without adequate funding, disability-inclusive healthcare policies remain largely symbolic (WHO 2018). While policies exist to promote disability inclusion, a lack of dedicated funding impedes their effective implementation (Republic of Namibia 2016; WHO 2018). A nationally centralised budget delays the execution of activities on disability affairs, which has a critical impact on the health outcomes of people with disabilities. These suggest a decentralised budget and an effective monitoring system to facilitate disability operations in remote settings.

Policymakers reported the existence of supportive policy documents, such as the *National Disability Act* and international conventions, as facilitators for accessibility to healthcare services for people with disabilities. Other studies have reported the importance of inclusive policies in reducing discrimination and emphasising the benefits of persons with disabilities (Jolley et al. 2018). However, participants in this study observed that laws and policies are gathering dust on their tables, resulting in weak policy enforcement and limited awareness among healthcare workers. These differences between policy and practice are a crucial issue that requires effective monitoring and education to ensure the smooth implementation of policies addressing the difficulties faced by people with disabilities (Gauthier-Beaupré et al. 2023; Jolley et al. 2018). The absence of remote monitoring and evaluation of duties hinders policy implementation to the detriment of disability matters. These findings suggest that supervisory visits should be prioritised to promote efficiency in rural areas when addressing disability issues.

Participants in this study emphasised the importance of prioritising people with disabilities in service delivery, particularly those with mobility challenges, so they are attended to first when queuing for health services. The existing services hinder the welfare of people with disabilities, as they lack reminders for follow-up appointments and delay prompt treatment. The findings challenge the recommended directives of the equity-based approach in healthcare that aims to provide preferential treatment for persons with disabilities (WHO 2022). On the other hand, the observed picture in this study reveals a lack of equity characterised by discrimination, which leads to poor-quality care. These findings challenge the global invitation to adopt corroborative policies at the national, local, and social welfare organisation levels, which conform to a holistic model of care, as endorsed by global health (Levesque, Harris & Russell 2013). Furthermore, intersectoral collaboration and the equitable promotion of healthcare are necessary to address complex health and social issues among diverse groups (Wang & Wilson 2022). There is a need for prioritised healthcare for disadvantaged groups, aiming to mitigate the consequences of inequality.

The findings also show training gaps among healthcare providers. Participants emphasised the need to train healthcare providers, particularly in sign language, to enhance communication. Many providers lack knowledge and skills related to disability affairs, which can lead to inadequate diagnosis and treatment for people with disabilities. This finding aligns with previous research that advocates for disability competency training across all health professions for positive health outcomes of people with disabilities (Belay 2020; Doody, Hennessy & Bright 2022; Sherry, Ned & Engelbrecht 2024). The continuing ignorance of disability affairs results in mistrust of service, poor adherence to treatment, with detrimental health effects to people with disabilities, which hinders the aim of Sustainable Development Goal Three and Universal Health Coverage. These suggested an inclusive teaching curriculum to address disability awareness and ensure accessibility for disadvantaged groups.

Participants reported negative attitudes and discriminatory behaviours among the healthcare providers in the care of people with disabilities. Stigma and discrimination in healthcare settings and family households were found to be pervasive, particularly towards people with intellectual and psychosocial disabilities. Previous studies have shown that stigma leads to social exclusion and poorer healthcare outcomes for people with disabilities, further establishing health disparities (Iezzoni et al. 2021; McClintock et al. 2018). Furthermore, the findings are in line with the work of others, indicating that stigma, especially regarding mental disorders, is common and deeply rooted in the community (Chou & Tseng 2020). This reflects broader societal attitudes that view people with disabilities as less capable or deserving of equal treatment. These findings indicate the need for inclusive policies and healthcare providers’ training to prevent the deteriorating health and adverse outcomes among people with disabilities.

### Strengths and limitations

A key strength of this study was policymakers’ discovery of barriers to effective healthcare access for people with disabilities, which requires a comprehensive approach to efficient service delivery in line with the *Universal Health Coverage and the National Disability Act*. This study is limited in that it identifies possible recommendations for policies but lacks a mechanism for follow-up or monitoring beyond this research. The lack of service users’ voices, specifically those of persons with disabilities, is another limitation that future research should investigate more deeply.

### Implications and recommendations

This study has implications for policy and practice regarding policy reforms, the implementation of health services interventions, and their monitoring across various sectors. There is a need to include disability in the training curriculum for healthcare professionals, with a focus on basic awareness, communication strategies, and ethical considerations to promote positive behaviours among healthcare providers.

The government must take a more proactive role in ensuring that disability-inclusive policies are adequately funded, implemented, and effectively monitored. There should be sufficient funding for accessible infrastructure, especially for mobility-challenged individuals, funds for staff training, and disability awareness outreach to destigmatise the perception of disability.

Future studies can utilise this study’s findings to investigate the factors that facilitate the implementation of inclusive policies. Proposed studies could also include the views of other stakeholders involved in disability affairs across various governmental and non-governmental agencies at the regional and national levels. Comparative studies can be used to develop databases of people with disabilities for alignment with health service provision.

## Conclusion

The study identified several barriers influencing access to health services for people with disabilities. Key obstacles included transportation challenges, inadequate infrastructure because of limited funding, weak policy enforcement, and a lack of awareness of disabilities among service providers. These factors contribute to stigma, discrimination, and social exclusion, ultimately leading to poorer health outcomes. There is a need to address the barriers through targeted training, stronger policy implementation, and sufficient funding to enhance inclusivity and equity in health service provision for people with disabilities.
